# Wastewater-based surveillance of norovirus GI and GII: Comparative performance of commercial and in-house RT-qPCR Assays

**DOI:** 10.1007/s42770-026-01939-3

**Published:** 2026-04-22

**Authors:** Renato Becho Moura, André Vinicius Costa Ribeiro, Mateus de Souza Mello, Camille Ferreira Mannarino, Fábio Correia Malta, Fernando César Ferreira, Tulio Machado Fumian, Marize Pereira Miagostovich

**Affiliations:** 1 Post-graduate Program in Public Health and Environment of the Sergio Arouca National School of Public Health (ENSP), Oswaldo Cruz Foundation (Fiocruz), Rio de Janeiro, RJ Brazil; 2 Department of Viral Vaccines of the Institute of Technology in Immunobiological (Bio-Manguinhos), Fiocruz, Rio de Janeiro, RJ Brazil; 3 Department of Sanitation and Environmental Health, Sergio Arouca National School of Public Health (ENSP), Fiocruz, Rio de Janeiro, RJ Brazil; 4 Laboratory of Comparative and Environmental Virology, Oswaldo Cruz Institute (IOC), Fiocruz, Rio de Janeiro, RJ Brazil

**Keywords:** Norovirus GI, Norovirus GII, Wastewater treatment plant, Raw sewage

## Abstract

Noroviruses, the leading cause of nonbacterial acute gastroenteritis outbreaks, are widely detected in wastewater worldwide and reflect community-level viral circulation. These RNA viruses exhibit high mutation and recombination rates, favoring the emergence of novel variants with epidemiological relevance. In this study, wastewater-based epidemiology was applied to monitor the circulation of norovirus genogroups GI and GII in raw sewage samples collected biweekly throughout 2024 from a wastewater treatment plant serving the Metropolitan Region of Rio de Janeiro, Brazil. In parallel, the analytical performance of a commercial RT-qPCR kit was evaluated in comparison with an in-house RT-qPCR protocol for norovirus detection and quantification. A total of 24 samples were concentrated by ultracentrifugation, and viral nucleic acids were extracted using the Quick-DNA/RNA™ Viral MagBead kit. Norovirus GI and GII were detected and quantified using both the IBMP Biomol Rotavirus and Norovirus Kit and in-house assays. The IBMP kit showed higher detection rates for GI (54.2%) and GII (100%) compared with the in-house protocol (20.9% and 70.8%, respectively), as well as higher median viral concentrations for both genogroups. [GI (5.8 ± 0.7 vs. 4.8 ± 0.2 log_10_ gc µL^-1^) and GII (7.1 ± 0.7 vs. 6.6 ± 0.8 log_10_ gc µL^-1^)]. Molecular characterization based on partial ORF1 (RdRp) and ORF2 (VP1) regions identified genotypes GII.17[P17], GI.3[P3], and GI.3[P13], indicating the environmental circulation of globally disseminated strains previously associated with outbreaks. Overall, these findings support the applicability of wastewater-based surveillance for monitoring norovirus genotype circulation and highlight the usefulness of a commercial RT-qPCR assay for sensitive detection and quantification of noroviruses in environmental samples.

## Introduction

*Norovirus*, a genus within the *Caliciviridae* family, comprises positive-sense, single-stranded RNA viruses and represents one of the leading causes of acute gastroenteritis (AGE) outbreaks worldwide [1]. According to the World Health Organization, noroviruses are responsible for an estimated 685 million cases of gastroenteritis annually, including approximately 200 million infections in children under five years of age. The global burden is substantial, contributing to nearly 200,000 deaths each year, about 50,000 of which occur among young children, and disproportionately affecting low-income countries [2].

Based on full viral protein 1 (VP1) gene sequences, noroviruses are classified into ten genogroups (GI–GX) and further subdivided into genotypes and variants, with more than 48 VP1-based genotypes and 60 RNA-dependent RNA polymerase (RdRp) (P-type) variants currently recognized [3–6]. Human noroviruses are associated with genogroups GI, GII, and GIV, with GI and GII being the most prevalent. The GII.4 genotype alone has accounted for 50–70% of outbreaks over the past decades, evolving seasonally, with dominant variants replacing previous strains every 2–4 years, including six pandemic GII.4 variants. In Asia, the recombinant GII.17[P17] genotype emerged in 2014–2015 and, together with GII.4 and its variants, has caused widespread outbreaks worldwide [3–6].

Due to its global burden and pandemic potential, the World Health Organization classified norovirus as a priority pathogen for vaccine development in 2016 [3, 7]. The global prevalence of norovirus gastroenteritis has remained consistently high since the emergence of the GII.4 Sydney 2012 variant, underscoring the urgent need for strengthened prevention and control strategies, particularly for children under five years of age and adults over 60 [8]. Although no licensed vaccine is currently available, several multivalent candidates targeting predominant genotypes (e.g., GII.4, GI.1) are under development. Their efficacy, however, may be influenced by factors such as pre-existing immunity, host age, and genetic background [9–11].

In this context, norovirus surveillance is critical for monitoring the circulation of genotypes and detecting emerging variants, which may have implications for vaccine development. Wastewater-based epidemiology (WBE) has proven to be a valuable tool for this purpose [3, 5, 9, 12–14]. WBE enables near real-time monitoring of viral dynamics and provides a complementary approach to capture community-wide viral circulation, including asymptomatic and pre-symptomatic infections that are often missed by clinical surveillance [15]. In this study, we applied WBE to investigate the occurrence, genotype circulation, and temporal dynamics of norovirus GI and GII in the Metropolitan Region of Rio de Janeiro, Brazil, while also evaluating the performance of a commercial RT-qPCR kit for their detection and quantification over a one-year surveillance period.

## Materials and methods

### Area, sample collection site and study period

This study was conducted in Niterói, a metropolitan municipality in the State of Rio de Janeiro. The municipality is divided into five administrative regions, covering a total area of 133.757 km², with approximately 481,749 residents and a population density of 3,601.67 inhabitants per km² [16] (IBGE, 2022). Sewage collection services cover 95.5% of the population, and 100% of the wastewater is treated [17].

Sewage samples were obtained from the main wastewater treatment plant (WWTP), which is responsible for treating 51% of the municipality’s total effluent and operates with a flow capacity of 1,350 L s^− 1^. The WWTP applies chemically assisted primary treatment using ferric chloride and discharges the treated effluent into the Icaraí submarine outfall [18].

From January to December 2024, biweekly sampling campaigns were conducted to collect composite raw sewage samples, accounting for daily variations in effluent characteristics. Samples were collected at the end of a sand removal tank, in sterile, labeled polypropylene bottles and transported in thermal boxes with ice packs. Procedures during sampling included the use of disposable gloves, keeping the bottles closed until the exact moment of collection, avoiding contact with the inner rim or the cap, and ensuring that the bottles were properly sealed. Upon arrival at the laboratory, the bottles containing the samples were stored at − 20 °C for a maximum period of 45 days prior to laboratory analyses. This period allowed the accumulation of up to six samples, corresponding to the maximum capacity of the ultracentrifuge rotor used for sample concentration. After concentration, the samples were processed for RNA extraction and subsequently stored at − 80 °C. Quantification of the samples for both methodologies was performed at the end of the one-year period.

### Viral concentration and RNA extraction method

Viral particles were concentrated from **42 mL of each sample** using the ultracentrifugation method previously described by Pina et al. (1998) [19]. Samples were processed in a Sorvall WX Ultra Series ultracentrifuge (Thermo Scientific, Waltham, MA, USA), using a Type 35 fixed-angle rotor followed by a SW 41 swinging-bucket rotor.

Prior to viral concentration, all samples were artificially spiked with 10 µL of bacteriophage PP7 containing 1.45 × 10⁷ genome copies. The PP7 bacteriophage strain was kindly provided by Dr. Verónica Rajal and was used as an internal process control (IPC) to monitor viral recovery and the efficiency of the concentration and nucleic acid extraction steps [20]. The amount of PP7 added was determined based on preliminary laboratory assays to ensure consistent detection by RT-qPCR while avoiding interference with the detection of the target viruses.

Nucleic acids were extracted from the concentrates using the Quick-DNA/RNA™ Viral MagBead kit (Zymo Research, Irvine, USA), according to the manufacturer’s instructions.

Positive fecal suspensions of norovirus GI and GII from the biorepository of the Regional Reference Center for Rotavirus, housed at the Laboratory of Comparative and Environmental Virology, were included as positive controls, while UltraPure™ DNase/RNase-free distilled water (Invitrogen, Carlsbad, CA, USA) was used as a negative control.

### TaqMan-base qPCR assays

For PP7 bacteriophage quantification a reverse transcription (RT) reaction was performed using the High-Capacity cDNA Reverse Transcription™ kit (Applied Biosystems^®^, Valencia, CA, USA), according to the manufacturer’s recommendations. Following, undiluted and 1/10 diluted samples were analyzed, both in duplicates to evaluate matrix inhibition using the Universal PCR Master Mix (Applied Biosystems), primers and probe previously described [19, 21].

For noroviruses, a Multiplex GI and GII in-house one-step RT-qPCR amplification reaction protocol was conducted using primers and probes targeting the junction region between open reading frame 1 (ORF1) and ORF2 [21].

TaqMan-base qPCR assays were performed using Applied Biosystems^®^ 7500 Real-Time PCR Systems thermocycler (both from Applied Biosystems, Foster City, CA, USA). All samples were analyzed together with a negative control (UltraPure™ DNase/RNaseFree distilled water), a no-template control (NTC), and standard curves (Integrated DNA Technologies, Coralville, Iowa, USA) with serial dilution at base 10 for each virus. Samples with a Ct (Cycle threshold) value ≤ 40 were considered positive.

The commercial Institute of Molecular Biology of Paraná (IBMP) Biomol Rotavirus and Norovirus kit was also used for the detection and quantification of norovirus GI and GII following the conditions of the manufacturer’s manual (IBMP, Paraná, Brazil 2024). It is a one-step multiplex kit that performs cDNA synthesis and has three viral targets (rotavirus, norovirus GI/GII). Although the kit presented results for rotavirus, it was not considered because it was not the scope of this study.

### Limit of detection (LoD) determination

Two complementary approaches were employed to estimate the LoD: (i) the assay-level LoD (ALoD), derived from the standard curve, and (ii) the process-level LoD (PLoD), which reflects the entire sampling and analytical workflow.

The ALoD was established using a 10-fold serial dilution of the standard curve, performed in duplicate, with concentrations ranging from 1.0 × 10¹ to 1.0 × 10⁶ gc µL^− 1^.

For the PLoD estimation, triplicate assays were conducted using RNA extracted from a sample previously confirmed as consistently positive for genogroups GI and GII with both the IBMP and in-house RT-qPCR protocols. This RNA was serially diluted to achieve theoretical concentrations from 1.0 × 10² to 1.0 × 10⁴ gc µL^− 1^.

Both ALoD and PLoD were estimated to be using a previously described logistic regression model [22] providing a robust and statistically valid determination of detection thresholds. Corresponding Ct values were calculated based on the standard curve’s slope, y-intercept, and respective quantification data.

### Norovirus genotyping

Norovirus-positive samples were subjected to conventional one-step RT-PCR for dual genotyping targeting the RdRp and VP1 genes as previously described [23, 24]. The reactions were performed using the Qiagen One Step RT-PCR kit (Qiagen) with primers Mon 432/G1SKR for GI and Mon 431/G2SKR for GII, that amplifies the ORF 1/2 junction region, generating PCR amplicon sizes of 543 and 557 base pairs, respectively (Table 1).

**Table 1:** Primers used for amplification of norovirus GI and GII targeting the ORF1–ORF2 junction

Genome region	Primer name	Sequence (5’ − 3’) *	References
ORF 1	Mon 431 (+)	TGG ACI AGR GGI CCY AAY CA	[23]
	Mon 432 (+)	TGG ACI CGY GGI CCY AAY CA	
ORF2	G1SKR (-)	CCA ACC CAR CCA TTR TAC A	[24]
	G2SKR (-)	CCR CCN GCA TRH CCR TTR TAC AT	

* Y = C/T, R = A/G, I = Inosine, N = A/C/T/G, H = A/C/T

The amplified products were analyzed by electrophoresis in 1% agarose gel at a voltage of 100 V for 40 min. The gel was developed using a GelDoc Go Imaging System^®^ transilluminator (Bio-Rad Laboratories, California, USA). Amplicons were purified using the commercial kit Wizard^®^ SV Gel and PCR Clean-Up System (Promega, Madison, MI, USA) following the manufacturer’s recommendations and sent to the Institutional DNA Sequencing Platform (PDTIS) of Oswaldo Cruz Foundation (Fiocruz) for Sanger sequencing in both directions using the commercial BigDye Terminator^®^ Kit and an ABI Prism 3730 Genetic Analyzer (Applied Biosystems, Foster City, CA, USA).

Consensus sequences were obtained from chromatogram using Geneious Prime 2021.1.1 software (Biomatters Ltd., Auckland, New Zealand). Sequences were genotyped using the norovirus typing tools (https://mpf.rivm.nl/mpf/typingtool/norovirus/and/https://calicivirustypingtool.cdc.gov/bctyping.html). In addition, consensual sequences were confirmed in terms of closest similarity sequence, using Basic Local Alignment Search Tool (BLAST).

Dendrograms were constructed based in partial RdRp and VP1 protein (ORF1 and ORF2, respectively) using the neighbor-joining method for GI and GII norovirus. The best substitution models were selected based on the corrected Bayesian Information Criterion (BIC) value as implemented in Molecular Evolutionary Genetic Analysis [25] and model used in this study was Kimura 2-parameter (K2) + G in both regions (2,000 bootstrap replications for branch support) [26]. Norovirus reference sequences were obtained from the National Center for Biotechnology Information (NCBI).

### Nucleotide sequence deposition

To ensure transparency and reproducibility, all nucleotide sequences generated in this study were deposited in the GenBank database. Norovirus GI sequences were submitted under accession numbers PX359222–PX359225 and PX359226–PX359227, while GII sequences were deposited under accession numbers PX359259–PX359267. The quality of nucleotide sequences was evaluated using the software Geneious to visually inspect and identify ambiguous nucleotides, sequencing artifacts, and insertions or deletions. Low-quality regions at the sequence ends were trimmed when necessary. Sequence integrity was also assessed by verifying the absence of stop codons or frameshifts within the expected open reading frame (ORF). In addition, sequence identity was confirmed by comparison with reference sequences available in public databases using the BLAST prior to phylogenetic analysis.

Furthermore, the sequences were checked for sequence length (~ 543 bp for norovirus GI and ~ 557 for GII) and integrity, before submission. All sequences were deposited and are publicly available for comparative and evolutionary analyses in the NCBI GenBank database [27].

### Statistical analysis

GraphPad Prism version 8 (GraphPad Software) and Dotmatics [28] were used for data processing and analysis. The Shapiro–Wilk test was applied to assess normality, while the Wilcoxon signed-rank test (paired data) and unpaired t-test were employed to compare viral loads and genotype distributions. Statistical significance was defined as a p-value ≤ 0.05.

## Results

### Internal control process

The PP7 bacteriophage was detected in 100% (24/24) of the samples with recovery efficiencies ranged from 33.3% to 108.2%.

### Limit of detection (LoD) for noroviruses

Standard curves were constructed for each RT-qPCR assay and genogroup to determine the limit of detection (LoD). The minimum LoD was estimated at 4.47 genomic copies per microliter (gc µL⁻1), with corresponding Ct cut-off values of 35.63 and 42.80 for in-house assay and 33.94 and 37.51 for IBMP kit (Table 2). The in-house assays showed amplification efficiencies of 91.2% for GI and 84.4% for GII, whereas the IBMP kit exhibited higher efficiencies (96.6% for GI and 103.3% for GII). Linearity was comparable between both methods.

**Table 2:** Quality, efficiency, and performance parameters of the qPCR assay in 24 samples according to the methodology used

Parameters	Methodologies/Genogroups			
	In House		IBMP	
	GI	GII	GI	GII
Slope	-3.552	-3.761	-3.381	-3.245
R2	0.99	0.98	0.97	1.00
Amplification efficiency	91.2%	84.4%	96.6%	103.3%
Cycle threshold	35.63	42.80	33.94	37.51
Detection rate % (n)	20.9% (5)	70.8% (17)	54.2% (13)	100% (24)
Median Log10 gc µL⁻1	4.77 ± 0.24	6.61 ± 0.76	5.8 ± 0.70	7.06 ± 0.71

**Fig. 1:**
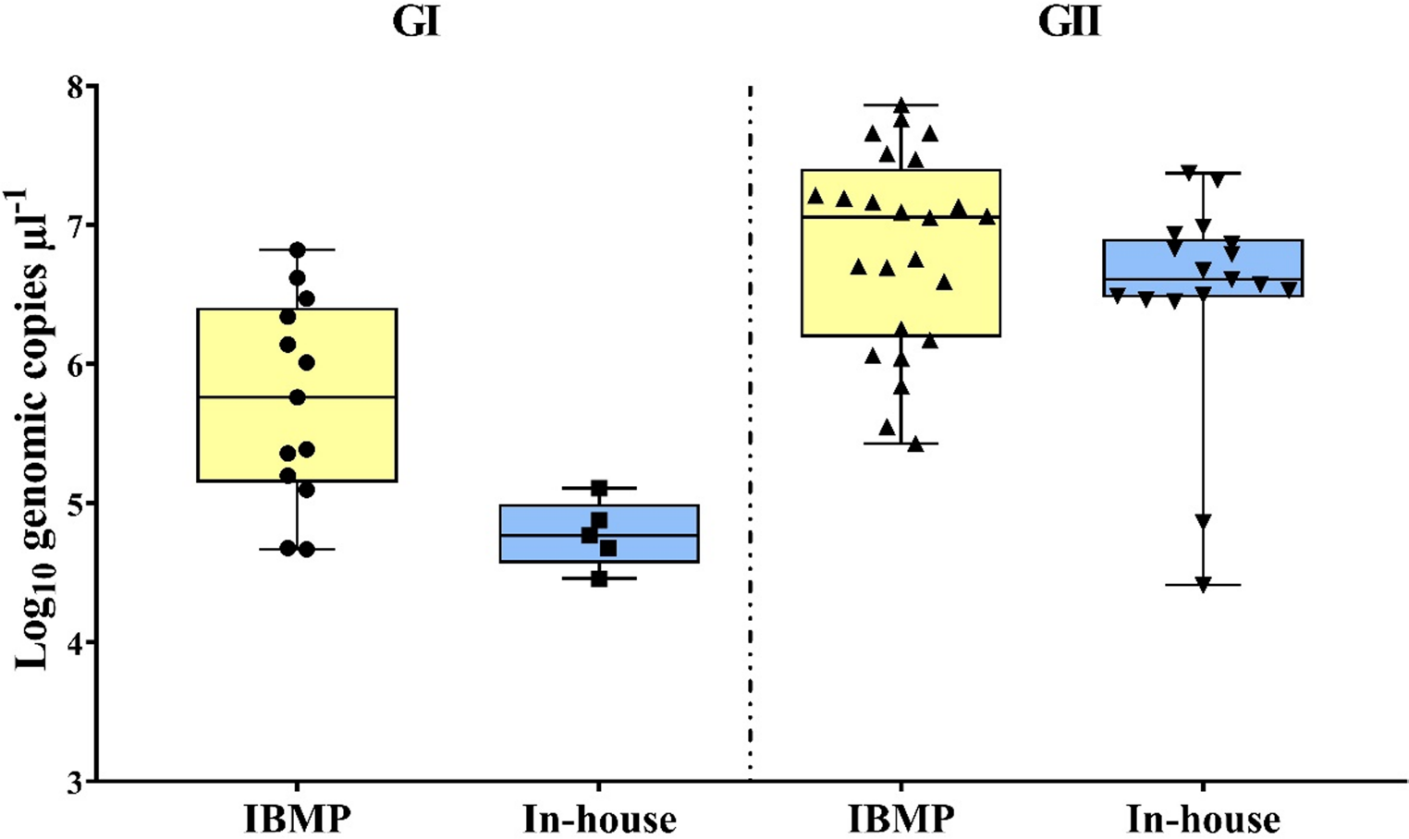
Norovirus quantification using the IBMP commercial RT-qPCR Kit and in-house protocols

The IBMP kit yielded higher median concentrations of norovirus GI and GII compared to the in-house protocol (Fig. 1). Although no statistically significant difference was observed for GI (*p* = 0.1250), a significant difference between the median concentration values was found for genogroup GII (*p* = 0.0001), indicating a higher sensitivity of the commercial kit in quantifying this genogroup.

**Fig. 2:**
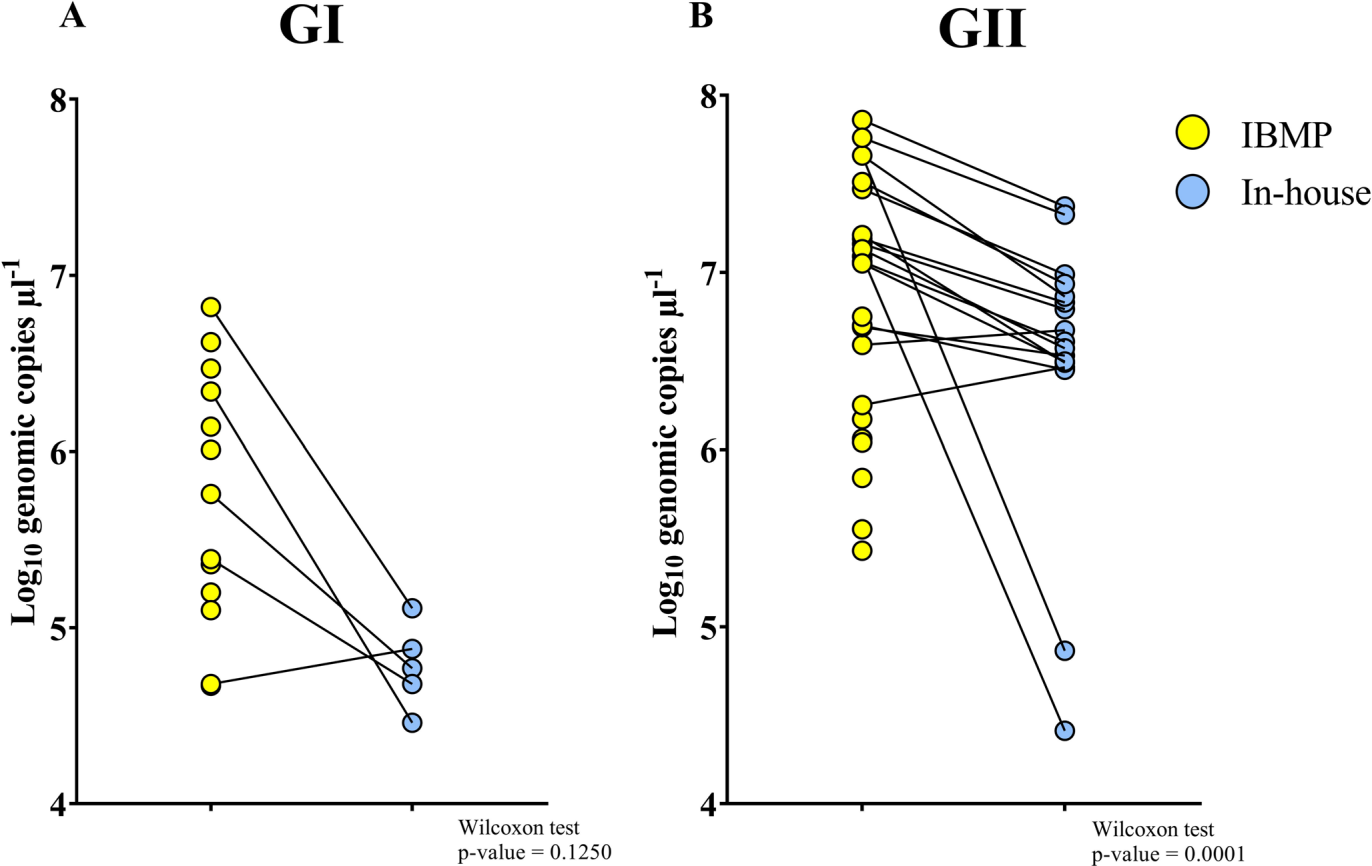
Paired comparison of norovirus RNA concentrations by protocol. Scatter plots showing individual norovirus GI and GII RNA concentrations measured by RT-qPCR using the IBMP kit and the in-house protocol. Each line connects paired values from the same sample, highlighting differences in quantification between methods

In the paired comparison of norovirus GII RNA concentrations obtained using the in-house RT-qPCR assay and the IBMP commercial kit, inter-sample variability in quantitative values was observed (Fig. 2). For a subset of samples, lower RNA concentration estimates were obtained with the in-house assay when compared with the commercial method. Paired statistical analysis using the Wilcoxon signed-rank test demonstrated a statistically significant difference between the two methods when all samples were considered (*p* = 0.0001). Importantly, this difference remained statistically significant (*p* = 0.0004) after exclusion of samples with lower quantification values, indicating that the observed difference was not driven by these observations and reflects a consistent difference in analytical performance between the assays. Preserving the full dataset allowed the representation of the inherent heterogeneity of environmental samples, a key feature of wastewater-based surveillance, and is consistent with the interpretation of the results under real-world conditions.

### Molecular characterization of noroviruses

**Fig. 3:**
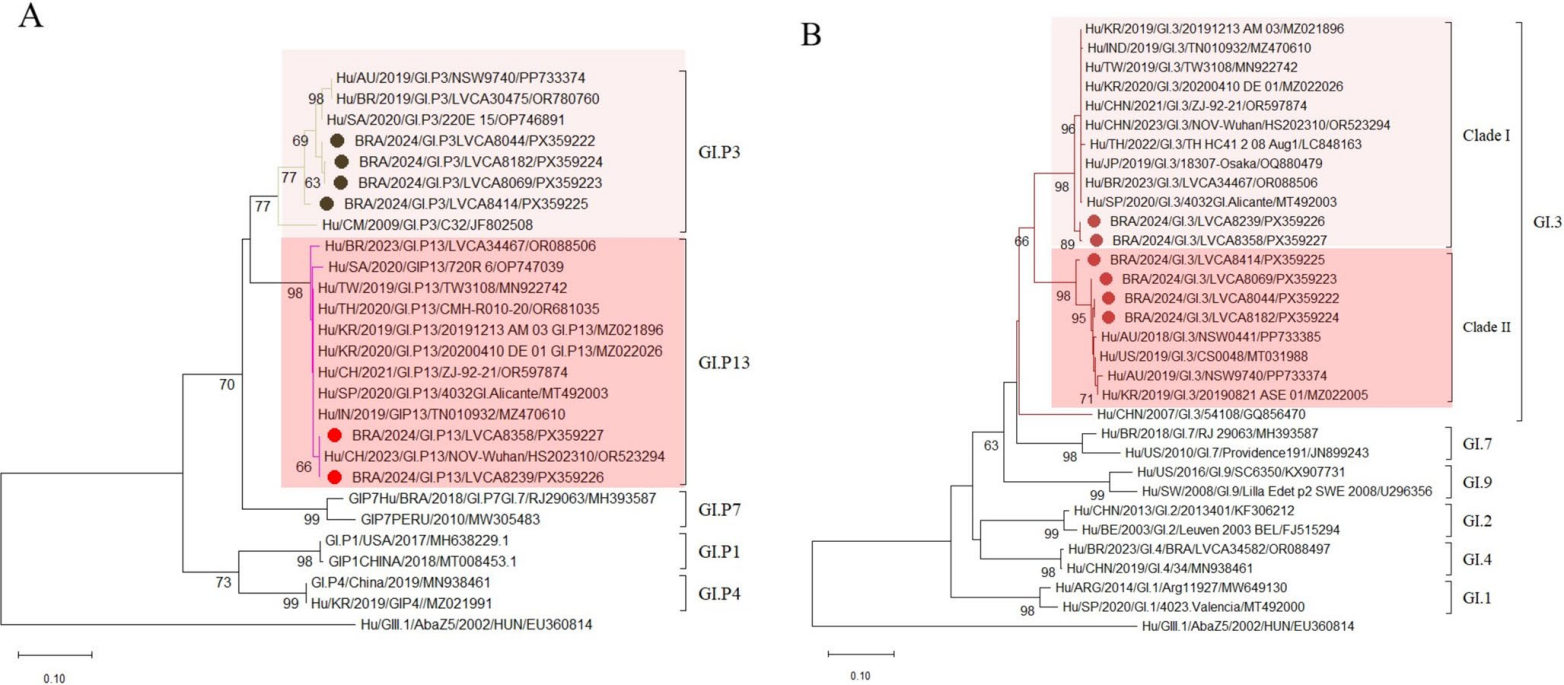
Characterization of norovirus GI genotypes. **A** based on the ORF1 (RNA-dependent RNA polymerase nucleotide sequence), **B** based on the ORF2 (capsid nucleotide sequence). Reference genotypes were obtained from GenBank and labeled with their respective data and accession numbers. The sequences obtained in this project (marked with a circle/diamond) were constructed with MEGA12 software and bootstrap (2000 replicates), based on the Kimura two-parameter model. They were identified by the abbreviation of the country of origin, followed by year of collection, genotype, the internal registration number and access number

**Fig. 4:**
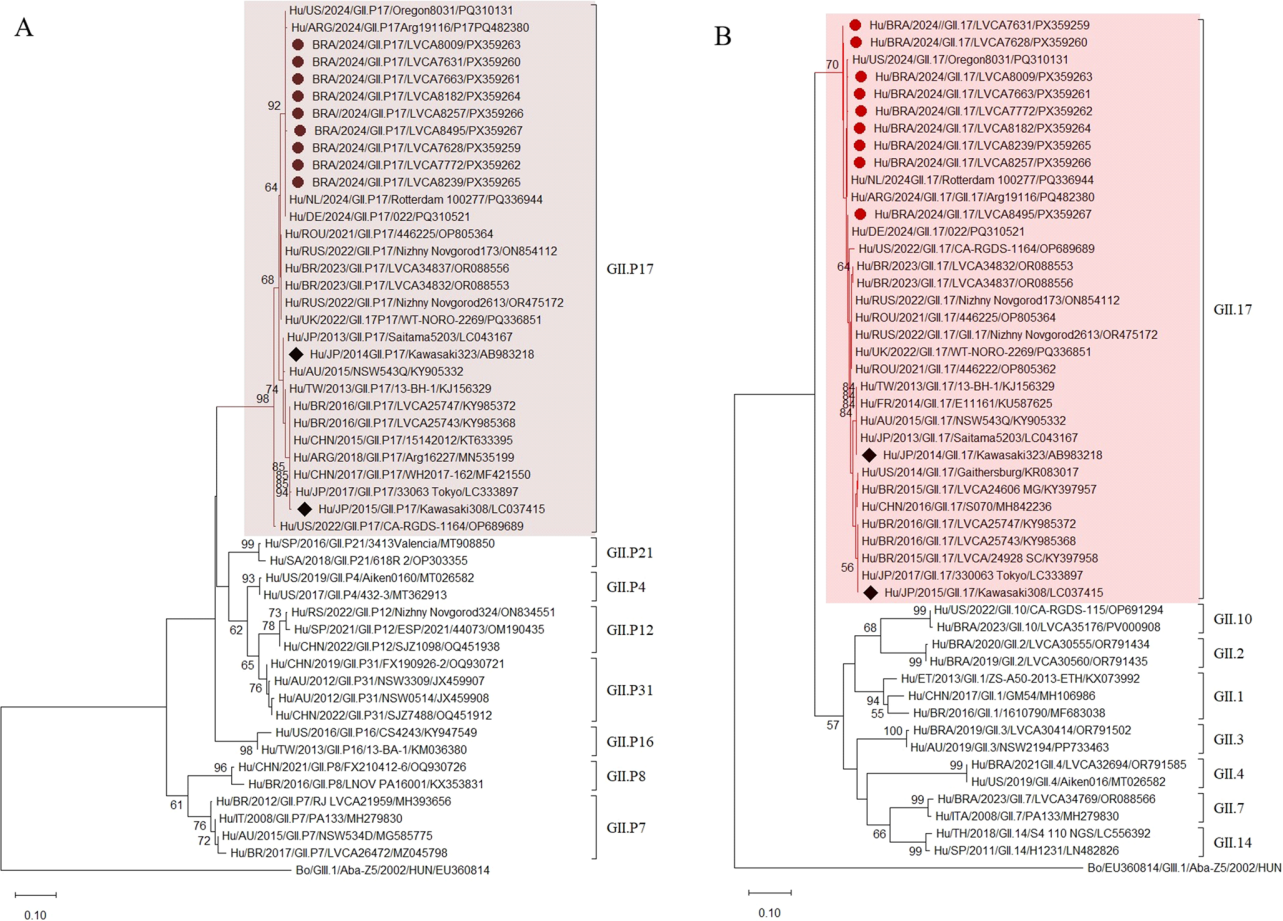
Characterization of norovirus GII genotypes. **A** based on the ORF1 (RNA-dependent RNA polymerase nucleotide sequence), **B** based on the ORF2 (capsid nucleotide sequence). Reference genotypes were obtained from GenBank and labeled with their respective data and accession numbers. The sequences obtained in this project (marked with a circle) were constructed with MEGA12 software and bootstrap (2000 replicates), based on the Kimura two-parameter model. They were identified by the abbreviation of the country of origin, followed by year of collection, genotype, the internal registration number and access number

Nucleotide sequencing analysis of the ORF1/ORF2 junction identified three norovirus genotypes: two from genogroup I (GI) and one from genogroup II (GII). The most prevalent was GII.17[P17], detected in nine samples. This was followed by GI.3[P3] (*n* = 4) and GI.3[P13] (*n* = 2) (Figs. 3 and 4).

Regarding GI, the polymerase region analysis revealed GI.P3 strains closely related showing nucleotide identities ranging from 97.2% to 99.6% among each other; except for one sample (BRA/2024/GI.P3/LVCA8414/PX359225), sequences formed a distinct phylogenetic cluster (Fig. 3A). GI.P3 strains formed a monophyletic branch and clustered separately from GI.P3 strains detected between 2019 and 2020 in Australia (PP733374), Brazil (OR780760), and South Africa (OP746891), as well as from a strain detected in Cameroon in 2002 (JF802508). Nucleotide sequence identities ranged from 88% to 98% (Fig. 3A).

GI.P13 strains exhibited 100% nucleotide identity with each other and with a sequence from China detected in 2023 (OR523294). They were closely related to previously reported strains from Brazil (OR088506/2023), Taiwan (MN922742/2019), South Korea (MZ021896/2019 and MZ022026/2020), China (OR597874/2021), South Africa (OP747039/2020), Thailand (OR681035/2020), Spain (MT492003/2020), and India (MZ470610/2019), sharing nucleotide identities ranging from 97.4% to 99.2% (Fig. 3A).

Analysis of the partial ORF2 (VP1) region identified the GI.3 genotype in association with polymerase types P3 and P13. GI.3 strains segregated into two distinct clades: clade I, comprising GI.3 strains associated with P13, and clade II, comprising GI.3 strains associated with P3. The two sequences within clade I were closely related to strains from South Korea (MZ021896/2019 and MZ022026/2020), India (MZ470610/2019), Taiwan (MN922742/2019), China (OR597874/2021 and OR523294/2023), Thailand (LC848163/2022), Japan (OQ880479/2019), Brazil (OR088506/2023), and Spain (MT492003/2020), sharing nucleotide identities ranging from 97.5% to 98.2%. Within clade II, Brazilian GI.3 sequences were closely related to strains from Australia (PP733385/2018 and PP733374/2019), the United States (MT031988/2019), and South Korea (MZ022005/2019), exhibiting nucleotide identities between 96% and 99% (Fig. 3B).

Regarding GII, a high nucleotide identity (98.9–99.6%) was observed between the GII.17[P17] strains identified in this study and those reported in 2024 in the United States of America (PQ31031/2024), Argentina (PQ482380/2024), the Netherlands (PQ336944/2024), and Germany (PQ310521/2024) (Fig. 4).

### Temporal distribution of noroviruses

**Fig. 5:**
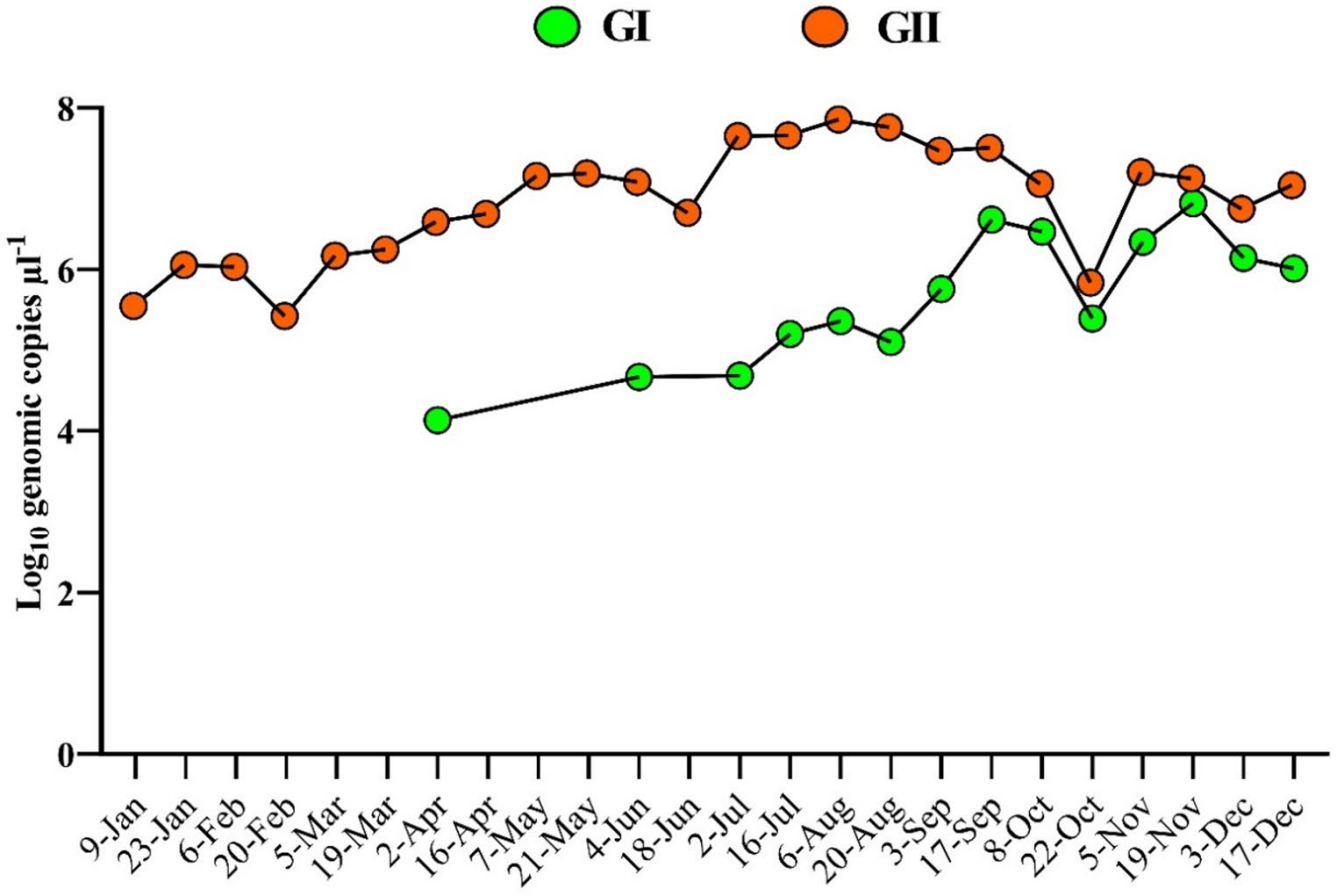
Quantification of norovirus. Genogroups I and II norovirus RNA levels were measured in bimonthly wastewater samples collected from WWTP between January and December 2024

Throughout the study period, genogroup GII was more prevalent than GI, both in detection frequency and in the number of genomic copies identified over the course of the year. Although GI noroviruses were detected in only two samples during the first half of 2024, their occurrence increased in the second half (Fig. 5). An unpaired t-test revealed a statistically significant difference between genogroups GI and GII (*p* < 0.0001). This analysis was performed using data obtained with the IBMP kit, as it demonstrated consistent analytical efficiency and sensitivity.

**Fig. 6:**
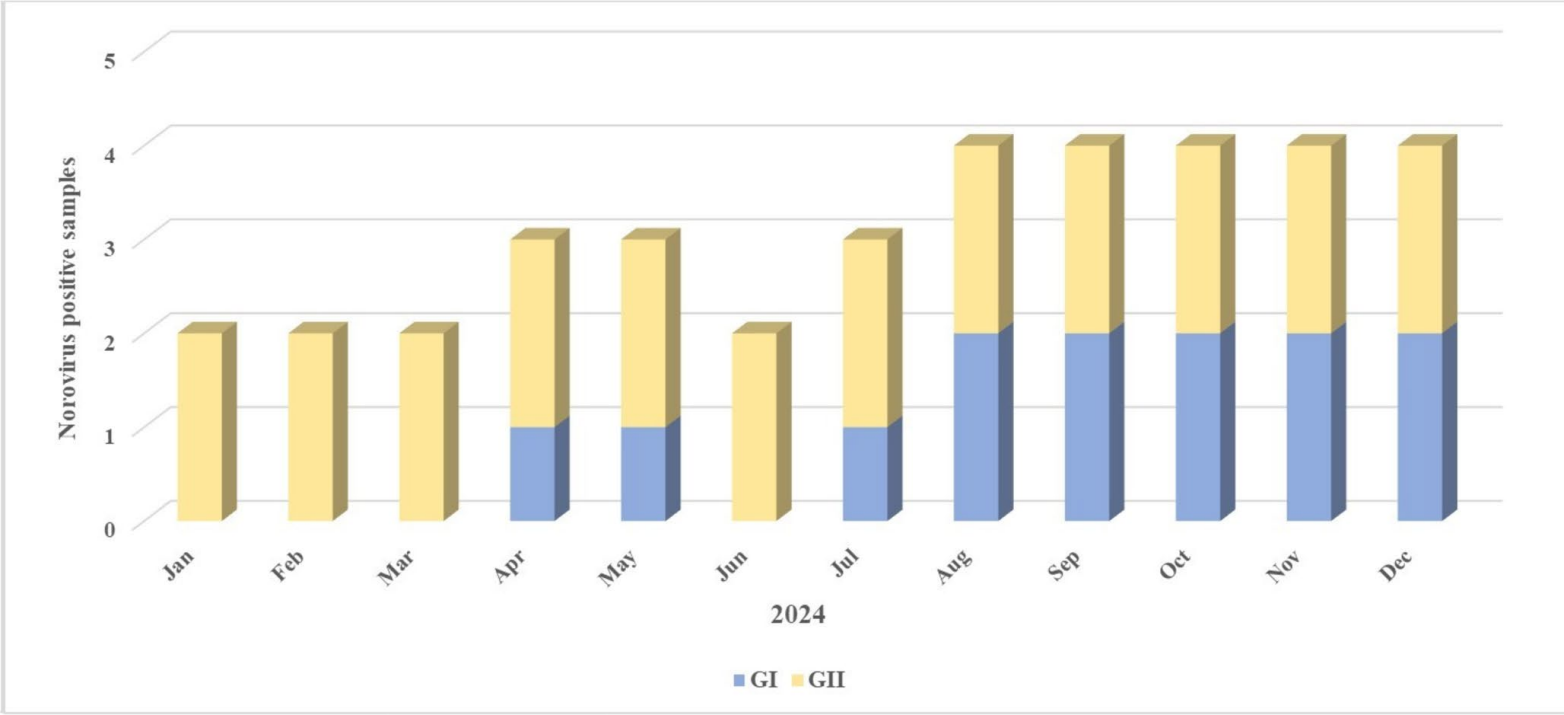
Monthly distribution of norovirus genotypes detected in Wastewater Samples – 2024

Regarding the temporal distribution of norovirus genotypes in wastewater samples, a predominance of GII.17[P17] was observed, particularly during the first and last quarters of the year. This genotype was detected from February to December, with peaks in February and September. In contrast, GI genotypes, specifically GI.3[P3] and GI.3[P13], were detected less frequently and mainly during the second half of the year, with co-circulation observed in September and December. Notably, GI.3[P13] was detected only in the final quarter (Fig. 6).

## Discussion

This study investigated the presence, RNA quantification, and molecular epidemiology of norovirus GI and GII through WBE at a WWTP in the municipality of Niterói, located in the metropolitan region of Rio de Janeiro. Three predominant genotypes were identified circulating during 2024. In addition, we successfully assessed the performance of the IBMP commercial RT-qPCR kit for the quantification of both genogroups in environmental samples, demonstrating its effectiveness when compared to an in-house RT-qPCR assay.

Originally developed as a qualitative assay for clinical diagnostics [29], the IBMP commercial kit was adapted in this study for application to wastewater samples by incorporating a standard curve for viral quantification. Its superior performance, compared to the in-house RT-qPCR protocol, was evidenced by an increased viral detection rate, potentially attributable to the use of optimized reagents, particularly in samples with low norovirus concentrations. The IBMP kit yielded higher median concentrations of norovirus GI and GII compared to the in-house protocol. Although the difference was not statistically significant for GI (*p* = 0.1250), a significant difference was observed for GII (*p* = 0.0001), indicating greater sensitivity of the commercial assay for this genogroup. The variability observed in norovirus GII RNA concentrations between methodologies reflect differences in assay sensitivity, reaction chemistry, or tolerance to matrix-associated inhibitors commonly present in wastewater samples. Considering that GII is the most frequently associated with human gastroenteritis outbreaks [3], improved detection may enhance the reliability of environmental surveillance. These findings suggest that optimized commercial assays may improve norovirus monitoring in environmental samples, particularly when viral concentrations are low or inhibitors are present.

Despite the recognized great diversity of polymerase types and capsid genotypes among noroviruses GI and GII, only three genotypes were identified during the study period. GII norovirus, particularly GII.17[P17], was the predominant genotype over the period. This finding aligns with the global epidemiological pattern and similar findings that have been reported in previous studies conducted in Brazil, South Africa, Thailand, and China [13, 30–32]. GII-positive samples also showed significantly higher RNA levels compared to GI-positive samples, consistent with other studies conducted in both environmental and clinical matrices [12]. In Shenzhen, China, wastewater surveillance [5] revealed higher noroviruses GII levels than GI and identified GII.4 (53.6%) and GII.17 (26.0%) as dominant genotypes, supporting its value for early outbreak detection.

Regarding temporal distribution, GII noroviruses were detected throughout 2024, with peak genomic concentrations during the winter months. Although seasonality has been well-documented in temperate regions, viral circulation in tropical areas is generally more homogeneous year-round [13, 33]. In this study, GI noroviruses were detected from April (early autumn), with increased frequency in the second half of the year, particularly associated with the emergence of the GI.3 genotype.

These findings highlight the importance of year-round wastewater-based surveillance, which can capture shifts in genotype prevalence and provide early warning signals of emerging strains, especially those with epidemic potential like GII.17[P17].

The exclusive detection of the GII.17[P17] genotype, predominantly during the early months of 2024, aligns with the observed predominance of GII.17 in clinical samples during the same period, as reported by the Reference Center (Dra. Burlandy F, Coordinator of the Brazilian Regional Reference Laboratory for Rotavirus and Norovirus, personal communication). The high sequence similarity between strains detected in this study and those circulating in recent outbreaks in Europe, Argentina, and the United States [4, 34] confirms their phylogenetic connection.

This emergent GII.17 variant, also known as the Kawasaki strain, first emerged in Japan between 2014 and 2015, spreading rapidly worldwide and often becoming predominant over other genotypes, at times surpassing GII.4 that has been the dominant norovirus genotype worldwide for over 20 years [6]. In Brazil, this strain has been detected in various matrices, including raw sewage, clinical samples, and bivalve mollusks intended for human consumption [30, 33, 35–37].

The predominance of the GI.3 genotype, particularly its P3 and P13 polymerase variants, is consistent with recent reports of GI.3 circulation in multiple countries, including Brazil, Japan, Taiwan, and South Africa [13, 30, 38, 39]. The phylogenetic analysis revealed two distinct clusters, with GI[P3] strains more closely related to GI[P7] than GI[P13]. These variants were also previously identified in Brazilian clinical samples between 2019 and 2022 [31].

The RT-PCR and Sanger sequencing strategy employed in this study detected viral strains present at higher concentrations, reflecting the dominant genotypes circulating in the population and may result in a lower apparent genotypic diversity compared to studies applying high-throughput sequencing techniques. For example, Fumian et al. (2019) [12], using MiSeq System, reported a broader diversity of norovirus genotypes in sewage samples, including a predominance of GII.4, a genotype not detected in the present study.

In conclusion, those findings highlight the utility of wastewater-based surveillance for monitoring norovirus circulation and support the applicability of the commercial RT-qPCR kit as a reliable tool for routine environmental surveillance. The IBMP kit showed superior sensitivity, higher amplification efficiencies, and increased detection rates, particularly for norovirus GII, resulting in higher quantified viral loads compared to the in-house protocol. The consistent detection of the PP7 internal process control in all samples confirmed the reliability of the analytical workflow. Moreover, the molecular characterization of outbreak-associated genotypes, with a predominance of GII.17[P17] and high genetic similarity to globally circulating strains, demonstrates that this approach provides reliable detection and characterization of epidemiologically relevant noroviruses.

## Data Availability

The data underlying this article are available in the article.

## Declarations

### Genetic heritage

This study was registered at the National System for the Management of Genetic Heritage and Associated Traditional Knowledge (SisGen) in compliance with the provisions of Brazilian Law N. 13123/2015 and its regulations, under numbers AB5FF8A and A6B4BFB.
